# Development of a Nationally Representative Built Environment Measure of Access to Exercise Opportunities

**DOI:** 10.5888/pcd12.140378

**Published:** 2015-01-22

**Authors:** Anne M. Roubal, Amanda Jovaag, Hyojun Park, Keith P. Gennuso

**Affiliations:** Author Affiliations: Anne M. Roubal, Amanda Jovaag, Hyojun Park, County Health Rankings and Roadmaps, University of Wisconsin Population Health Institute, Madison, Wisconsin.

## Abstract

We sought to develop a county-level measure to evaluate residents’ access to exercise opportunities. Data were acquired from Esri, DeLorme World Vector (MapMart), and OneSource Global Business Browser (Avention). Using ArcGIS (Esri), we considered census blocks to have access to exercise opportunities if the census block fell within a buffer area around at least 1 park or recreational facility. The percentage of county residents with access to exercise opportunities was reported. Measure validity was examined through correlations with other *County Health Rankings & Roadmaps*’ measures. Included were 3,114 of 3,141 US counties. The average population with access to exercise opportunities was 52% (range, 0%–100%) with large regional variation. Access to exercise opportunities was most notably associated with no leisure-time physical activity (*r* = −0.47), premature death (*r* = −0.38), and obesity (*r* = −0.36). The measure uses multiple sources to create a valid county-level measure of exercise access. We highlight geographic disparities in access to exercise opportunities and call for improved data.

## Background

Regular exercise participation is associated with lowered risk of premature mortality and chronic health conditions (1). Despite this knowledge, exercise participation remains low. Less than half of US adults meet the current guidelines for moderate-to-vigorous physical activity (≥150 min/wk of moderate, 75 min/wk of vigorous physical activity, or a combination) (2), and participation varies by region (3) with less physical activity in southern and western states.

Understanding why exercise participation varies by location must include an examination of the built environment (2,4). Most people exercise in their neighborhood, making accessibility to locations for exercise especially important (5). Those with gyms, neighborhood parks, or other recreational facilities in close proximity to their homes or work places are more likely to exercise (6–8). Although many different methods of systematically measuring the built environment exist, there is little consensus on which is the most appropriate.

The choice of a built environment measure for exercise is difficult because no standard measure exists. Giles-Corti et al (9) and Rutt (10) measured the distance from individual homes to the nearest recreational facility while others, such as Dill (11), focused on transit measures. Recreational facility density (12) or the ease of walking to specific locations such as parks (Walk Score) have also been used (13,14). Other methods, such as the ParkScore, which estimates the percentage of the population who are within a 10-minute walk of a well-maintained park, have been used as well, making a consensus for a comparable measure at different geographic levels difficult (15). Because of this lack of consensus, a new measure of access to exercise opportunities, measured at the county level, with national coverage, was created for the annual release of 2014 *County Health Rankings & Roadmaps* (www.countyhealthrankings.org) to address this research gap.

The *County Health Rankings* ranks counties within states on their current (Health Outcomes) and potential (Health Factors) health. One Health Factor area examined in the *Rankings* is Diet and Exercise. This Health Factor area includes measures of obesity, physical inactivity, and the food environment. We felt it was important to highlight the role that the built environment plays in providing opportunities for exercise. So, we developed a measure intended to provide communities a starting point for discussions on improving their county’s infrastructure to enhance exercise opportunities. In 2014, the *County Health Rankings* introduced the access to exercise opportunities measure, providing a nationally available county-level measure. The measure incorporates both park and recreational facility data to create a comparable county-to-county measure of access and the first to adjust for the differing needs and transportation patterns between urban and rural areas. We describe the development of the access to exercise opportunities measure, available at www.countyhealthrankings.org, and provide descriptive results.

## Methods

### Measure definition

The *County Health Rankings*’ access to exercise opportunities measure identifies the percentage of individuals in a county who live in reasonable proximity to a location for exercise. Exercise locations were split into 2 categories: parks and recreational facilities. Parks were defined as public land intended for recreational purposes and include land designations such as parks, forests, and wilderness areas. Recreational facilities were defined as establishments primarily engaged in fitness, recreational sports, exercise, or physical fitness conditioning.

Individuals who reside in a census block within one-half mile of a park, 1 mile of a recreational facility in urban areas, or 3 miles of a recreational facility in rural areas were considered to have access to exercise opportunities. These buffer distances correspond to what we believe individuals would walk to visit a park or drive to visit a recreational facility and are intended to approximate a 10-minute trip. This is in consideration of evidence to suggest that trips longer than 10 minutes are associated with increased odds of inactivity (16).

### Data

Park data were aggregated from 2 sources. Data were purchased from the DeLorme World Vector (MapMart) team and publicly accessible data were downloaded at no cost from the Esri online mapping database. The DeLorme MapMart and Esri geographic information system (GIS) data provide 2010 geocoded, projected data on parks at the local, state, and national level across the United States.

Recreational facilities were downloaded from OneSource Global Business Browser (Avention) and defined according to the North American Industry Classification System (NAICS) code 713940 (ie, establishments primarily engaged in operating fitness and recreational sports facilities featuring exercise and other active physical fitness conditioning), and include various facilities such as gyms, community centers, YMCAs, dance studios, and pools (17). This data set reflects all 2012 businesses classified as recreational facilities (Box).

**Box. Complete List of Lands or Facilities Included as a Park or Recreational Facility in the Access to Exercise Opportunities Measure**

**The following land designations are included as parks**:

- Bureau of Land Management Wilderness Area
- Campground
- County Park or Forest
- County Park or Forest on Island
- Geologic Area and/or Archaeological Site
- Miscellaneous Park in Indian Reservation
- Miscellaneous Park in Indian Reservation on Island
- Miscellaneous Park on Island
- National Environment Area
- National Environment Area on Island
- National Forest
- National Forest Wilderness Area
- National Forest on Island
- National Park
- National Park Wilderness Area
- National Park on Island
- National Recreational Area
- National Recreational Area on Island
- National Wildlife Management Area on Island
- National Wildlife Management Area
- National Wildlife Refuge
- National Wildlife Refuge on Island
- National Forest Wilderness Area on Island
- National Park Wilderness Area on Island
- State Environment Area
- State Environment Area on Island
- State Forest
- State Forest on Island
- State Park
- State Park on Island
- State Recreational Area
- State Recreational Area on Island
- State Wildlife Management Area on Island
- State Wildlife Management Area
- State Wildlife Refuge
- State Wildlife Refuge on Island
- Town or Local Park
- Town or Local Park on Island

**The following North American Industry Classification System codes were included as recreational facilities:**

- All Other Miscellaneous Schools and Instruction
- Child and Youth Services
- Civic and Social Organizations
- Diet and Weight Reducing Centers
- Fine Arts Schools
- Fitness and Recreational Sports Centers
- Freestanding Ambulatory Surgical and Emergency Centers
- Golf Courses and Country Clubs
- Legislative Bodies
- Nature Parks and Other Similar Institutions
- Offices of Mental Health Practitioners (except Physicians)
- Other General Government Support
- Other Individual and Family Services
- Other Social Advocacy Organizations

Population data were obtained from the US Census Topologically Integrated Geographic Encoding and Referencing (TIGER)/Line Files for the 2010 census at the census block level. These spatial data are available for download on the census website and were joined with American Community Survey demographic data (18).

### Measure calculation

First, using ArcGIS (Esri), buffers of one-half mile were created around parks identified from the Esri and DeLorme data sets. The buffer process was repeated using 1-mile buffers in urban and 3-mile buffers in rural areas around recreational facilities identified from the OneSource Global Business Browser data set. Urban/rural status at the census-tract level was determined by using ordinal data from the US Department of Agriculture Food Environment Atlas (19). All census blocks within a census tract were assigned the same urban/rural code. Counties without information on parks or recreational facilities in any of the 3 data sets were assigned missing values (n = 30).

The newly created buffer files were intersected with US Census TIGER/Line Shapefiles to assign census block population data (18). To identify census blocks with access to exercise opportunities, an indicator variable was created for census blocks where at least 1 of the buffered locations for exercise overlaps the census block. Then census block files were aggregated to a county level to obtain the final measure using SAS version 9.3 (SAS Institute, Inc). The final measure is the percentage of individuals in a county with access to an exercise opportunity. The formula used for the calculation was as follows:

[(Sum of Census Block Populations With Access to an Exercise Opportunity) ÷ (Sum of All Census Block Populations)] × 100

### Analyses

Construct validity of the access to exercise opportunities measure was explored using Spearman rank correlations to examine its association with other *County Health Rankings* measures sharing similar theoretical constructs. These included premature mortality (years of potential life lost before age 75), 3 quality-of-life measures (percentage of adults reporting fair or poor health and the average number of physically and mentally unhealthy days reported in the past 30 days), obesity (percentage of adults that report a body mass index ≥30, measured as weight in kg divided by height in m^2^), and physical inactivity (percentage of adults reporting no leisure-time physical activity in the past 30 days). Additional demographic analyses investigated potential associations between access to exercise opportunities and urban/rural area or income. The access to exercise opportunities measure was also aggregated to US state and census region to facilitate comparisons across the country.

## Results

### Descriptive statistics

**Figure 1:**
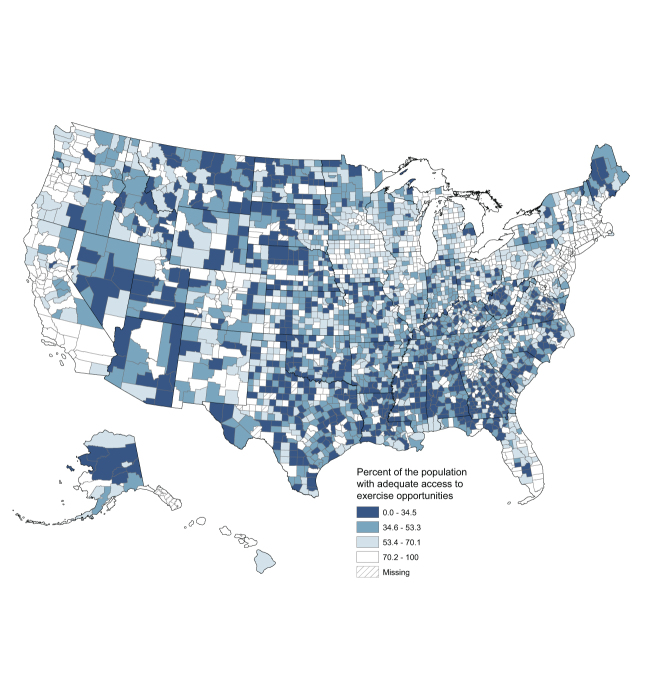
County-level distribution of access to exercise opportunities.

**Figure 2:**
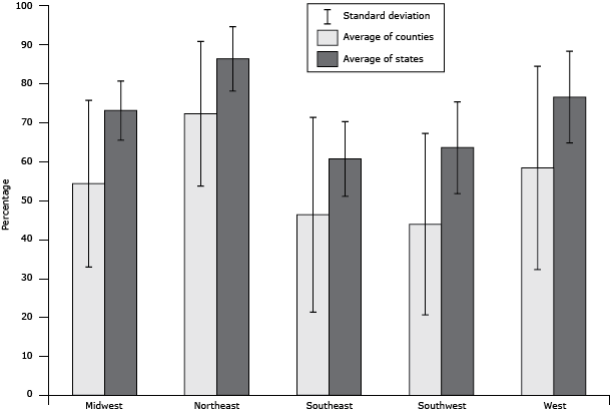
Regional variation of access to exercise opportunities. The average of states was calculated by using the mean values of state estimates for access to exercise opportunities for each region. The average of counties was calculated by using the mean values of county estimates for access to exercise opportunities for each region. The only significant difference was between counties in the Northeast and the Southeast (*P* < .05). **Table:** 

For the 2014 *County Health Rankings* the access to exercise opportunities measure was calculated for 3,114 of 3,141 counties. Access to exercise opportunities at the county level ranged from 0 (19 counties) to 100% (51 counties) with a mean of 52% and a standard deviation of 24.5%. The mean county population living within a half-mile of a park was 19.5% (range, 0%–100%), and the mean county population living within a mile of a recreational facility in urban (or 3 miles in rural) areas was 38.9% (range, 0%–100%). Figure 1 depicts population quartiles of access to exercise opportunities. The top 10% of counties had accessibility levels of 85% or greater. Statewide access varied from 46% (Mississippi) to 91% (Maryland); 100% of District of Columbia residents had access. Greater access to exercise opportunities occurred in northeastern (86%) and western (77%) states compared with southwestern (64%) and southeastern (61%) states (Figure 2). The national percentage of the population with access to at least 1 park or recreational facility was 77%.

Region	Percentage (Standard Deviation) of Counties	Percentage (Standard Deviation) of States
Midwest	54.4 (21.4)	73.1 (7.6)
Northeast	72.3 (18.6)	86.4 (8.3)
Southeast	46.4 (25.0)	60.7 (9.6)
Southwest	44.0 (23.3)	63.6 (11.8)
West	58.4 (26.1)	76.6 (11.8)

### Construct validity

The access to exercise opportunities measure was associated with several Health Outcome and Health Factor measures. It correlated most strongly with the percentage of adults reporting no leisure-time physical activity (*r* = −0.47) in the expected direction where counties with greater access to exercise opportunities had a smaller percentage of individuals reporting no physical activity. Access to exercise opportunities was also moderately associated with premature death (*r* = −0.38), percentage of adults reporting fair or poor health (*r* = −0.32), and obesity (*r* = −0.36). Weaker correlations were found between access to exercise opportunities and the average number of physically (*r* = −0.24) and mentally (*r* = −0.14) unhealthy days reported in the last 30 days. The measure was also significantly correlated with demographic indicators of geography (percentage of the population living in a rural area, *r* = −0.55) and income (percentage of children living in households with income below poverty thresholds, *r* = −0.29). All correlations were significant at the *P* < .01 level.

## Discussion

Links between the built environment and exercise participation have led to the development of various built environment measures, comprehensively reviewed by Brownson et al (20). We were unable to find a national measure using publicly accessible data for all counties in the United States before the 2014 *County Health Rankings*. Therefore, we developed the access to exercise opportunities measure using ArcGIS and multiple data sources to describe the percentage of the population living in close proximity to parks and recreational facilities. The ultimate goals of the measure were to provide county-to-county comparisons of the built environment for exercise and assist in discussions on the alteration of the built environment to promote exercise.

Overall, we found evidence to suggest that the access to exercise opportunities measure has good construct validity. It was significantly associated with several theoretically similar *Rankings*’ Health Factor and Health Outcome measures in the expected directions. Negative correlations were found with premature mortality, poor quality of life, and obesity, and the strongest correlation was with a lack of self-reported leisure-time physical activity. This indicated the access to exercise opportunities measure was performing in the intended manner.

We discovered significant variation when we used the measure to examine state and regional differences in access to exercise opportunities. There was regional variation characterized by the Northeast having the largest percentage of their population with access to exercise opportunities while the Southeast had the smallest. In the southern regions, less than 50% of county populations had access to exercise opportunities. This lack of opportunity is ecologically consistent with the lack of self-reported physical activity in southern states (3) and may help partially explain this phenomenon. There was also large within-region variation evident by large standard deviations at the county level (Figure 2). Even within states, county variation and disparity in access exist. For example, in Wisconsin, only 1% of the population in Menominee County has access to an exercise opportunity compared with 98% of the population in Milwaukee County.

This measure is intended to initiate discussions on the relationship between the built environment, exercise, and health. Public health officials and community leaders can view the percentage of the population in their community with access to exercise opportunities and compare it to other counties at www.countyhealthrankings.org. This measure highlights public health disparities in terms of access to parks and recreational facilities. One obvious intervention to improve access to exercise opportunities is designating land for public parks. Parks can provide numerous physical, mental, and social health benefits for residents of a community (21). Additionally, they remove a significant exercise barrier if location efficiency is maximized so that a large percentage of the population lives close in urban areas (16) or if co-located near other amenities such as churches, town halls, or stores in rural areas. Accordingly, numerous studies have linked park use to increased likelihood of sufficient physical activity levels (22,23). Another public health intervention is to encourage the development of recreational facilities such as gyms, YMCAs, and community centers with exercise components. Several studies have found positive associations between use of these types of facilities and exercise. The likelihood of meeting physical activity recommendations was similarly increased by use of a private facility (22), access to an indoor facility (24), and use of an indoor gym (25).

The access to exercise opportunities measure serves as the first national measure, to our knowledge, of the built environment combining park and recreational facility information at the county level. It provides for all 50 states and 3,114 of 3,141 counties an objective assessment of their built environment for exercise. It also provides a method for comparison across counties or states, allowing communities to gauge their access relative to their peers. Further, this measure will change as parks and recreational facilities are added or removed or when population shifts occur, so longitudinal trends analysis and the evaluation of interventions to improve access are possible. The main source of strength for the access to exercise opportunities measure is the use of GIS tools. This allowed us to create a measure that incorporates multiple locations for exercise from several data sources on a large geographic scale. Researchers and public health practitioners can tailor the measure to their local geographic needs by adjusting buffer distances and adding data from other sources unavailable at the national level.

There are several limitations inherent in creating a measure on a national scale using archival data. First, no data set accurately captures data on all the possible locations for exercise within a county. One notable location not included in this measure is sidewalks, common locations for running or walking. Additionally, not all locations for exercise are identified by their primary or secondary business code. For example, malls frequently have walking clubs and schools may have open gyms for community members. Second, although a county may contain a park or recreational facility, there may still be barriers, such as cost and distance, to using the facility. Locations for exercise, as defined by using NAICS secondary codes, may slightly overestimate access to exercise opportunities. This measure also did not capture data on other barriers to the accessibility of parks or recreational facilities for exercise. The size or amenities of parks clearly differs across the data set, and we were unable to adjust for hours of operation, entrance locations, fees for entrance to parks, or membership fees for gyms and exercise classes. Finally, there are potential inaccuracies in the data sources because they are from previous years and may not capture data on all of the opportunities for exercise.

The 2014 *County Health Rankings* included a measure of opportunities for exercise that is comparable across counties. Although the access to exercise opportunities measure is not all-inclusive, it is the first to combine parks and recreational facilities to create a county-level measure. This measure should serve as a call for researchers and public health officials to engage in activities related to enhancing the built environment for exercise, such as improving and maintaining parkland, reducing gym membership fees, or providing organized community recreation programs. With such activities, our understanding of how the built environment influences health will advance and lead to public health interventions to improve health for all.
